# In Situ Growth of Sodium Manganese Hexacyanoferrate on Carbon Nanotubes for High-Performance Sodium-Ion Batteries

**DOI:** 10.3390/molecules29020313

**Published:** 2024-01-08

**Authors:** Can Guo, Jianxiong Xing, Ali Shamshad, Jicheng Jiang, Donghuang Wang, Xin Wang, Yixuan Bai, Haifeng Chen, Wenwu Sun, Naying An, Aijun Zhou

**Affiliations:** 1 School of Materials and Energy, University of Electronic Science and Technology of China, Chengdu 611731, China; 2 Yangtze Delta Region Institute (Huzhou), University of Electronic Science and Technology of China, Huzhou 313001, Chinawdh@csj.uestc.edu.cn (D.W.); wangxin@csj.uestc.edu.cn (X.W.); 3 Huzhou Key Laboratory of Green Energy Materials and Battery Cascade Utilization, School of Intelligent Manufacturing, Huzhou College, Huzhou 313000, China; 4 Thermo Fisher Scientific Co., Ltd., Building A, China Core Technology Park, 2517 Jinke Road, Pudong New Area, Shanghai 201206, China

**Keywords:** in situ growth, sodium manganese hexacyanoferrate, coprecipitation, electron transport, crystal water

## Abstract

Sodium manganese hexacyanoferrate (NaMnHCF) has emerged as a research hotspot among Prussian blue analogs for sodium-ion battery cathode materials due to its advantages of high voltage, high specific capacity, and abundant raw materials. However, its practical application is limited by its poor electronic conductivity. In this study, we aim to solve this problem through the in situ growth of NaMnHCF on carbon nanotubes (CNTs) using a simple coprecipitation method. The results show that the overall electronic conductivity of NaMnHCF is significantly improved after the introduction of CNTs. The NaMnHCF@10%CNT sample presents a specific capacity of 90 mA h g^−1^, even at a current density of 20 C (2400 mA g^−1^). The study shows that the optimized composite exhibits a superior electrochemical performance at different mass loadings (from low to high), which is attributed to the enhanced electron transport and shortened electron pathway. Surprisingly, the cycling performance of the composites was also improved, resulting from decreased polarization and the subsequent reduction in the side reactions at the cathode/electrolyte interface. Furthermore, we revealed the evolution of potential plateau roots from the extraction of crystal water during the charge–discharge process of NaMnHCF based on the experimental results. This study is instructive not only for the practical application of NaMnHCF materials but also for advancing our scientific understanding of the behavior of crystal water during the charge–discharge process.

## 1. Introduction

Renewable energy resources like solar energy and wind energy are abundant in nature; however, their decentralized distribution leads to an intermittent power supply. These challenges could be addressed by integrating energy storage systems [1,2]. Currently, lithium-ion batteries (LIBs) are the predominant electrochemical energy storage system applied for the development of renewable energy [3]. Nevertheless, the global availability of Li resources is limited and unevenly distributed, posing a challenge for future energy storage demands. Although the capacity of sodium-ion batteries (SIBs) is lower than that of LIBs, the abundant and widespread availability of sodium resources on earth make SIBs a promising option for a wide range of applications [4,5].

The larger ionic radius of the Na ion compared to the Li ion makes it infeasible to directly apply LIB materials to SIBs [6]. Therefore, there is a need to explore new cathode materials for SIBs. Since Neff reported the electrochemical activity of Prussian blue analogues (PBAs) in 1978 [7], PBAs have been recognized as suitable cathode materials for batteries [8,9]. Cui et al. employed PBAs as a cathode material in an aqueous sodium-ion battery in 2011 [10], demonstrating that PBAs hold great promise in the application of sodium-ion batteries due to their open double perovskite structural frameworks, fast kinetics, and decent specific capacity [11,12]. The general formula of PBAs is A_2-x_M[M’(CN)_6_]_1-y_ □ _y_·nH_2_O, where A represents an alkali metal site, M and M’ are transition metal sites, □ signifies [M’(CN)_6_]^4−^ vacancy, and H_2_O represents crystal water [13,14]. Among the many PBAs used as SIB cathode materials, Na_2-x_Mn[Fe(CN)_6_]_1-y_□_y_·nH_2_O (NaMnHCF) stands out with multiple advantages, namely, high specific capacity, high voltage plateau, low cost, and abundant raw materials [15]. However, there are challenges that need to be addressed to bring NaMnHCF into daily life. Inherently, NaMnHCF contains crystal water, which can negatively impact the performance of SIBs. Furthermore, the high vacancy content of [Fe(CN)_6_]^4−^ is also considered detrimental to the performance of SIBs [16]. In addition, other factors such as the Jahn–Teller effect of Mn^3+^ [17], the dissolution of Mn^2+^ [18], and the low electronic conductivity of NaMnHCF further compromise the electrochemical performance [19]. Compared with other challenges, much less attention has been paid on improving the electronic conductivity of NaMnHCF.

To improve the electronic conductivity of NaMnHCF, Jiang et al. [20] synthesized NaMnHCF using a coprecipitation method and then coated its surface with a layer of conductive polypyrrole (ppy) to obtain NaMnHCF@ppy composites. Ppy coatings not only serve to stabilize the crystal structure but also enhance the electrical conductivity of the material. Similarly, Jiang et al. [21] synthesized an RGO-coated Na_1.83_Ni_0.12_Mn_0.88_Fe(CN)_6_ material in one step using a facile coprecipitation method. The cladding layer also increases the surface conductivity and stabilizes the crystal structure of the material. Goodenough et al. [22] obtained a NaFeHCF/CNT composite structure, with CNTs traversing the NaFeHCF particles by adding pretreated CNTs during the synthesis of NaFeHCF through a single iron source method. The NaFeHCF/CNT composite structure shortens the electronic transport pathway of the cathode electrode, effectively accelerating the charge rate of SIBs [23]. The material has excellent cycling performance and multiple advantages, but the synthesis process of the single iron source method generates hazardous hydrogen cyanide, which is not conducive to large-scale production.

**Scheme 1:**
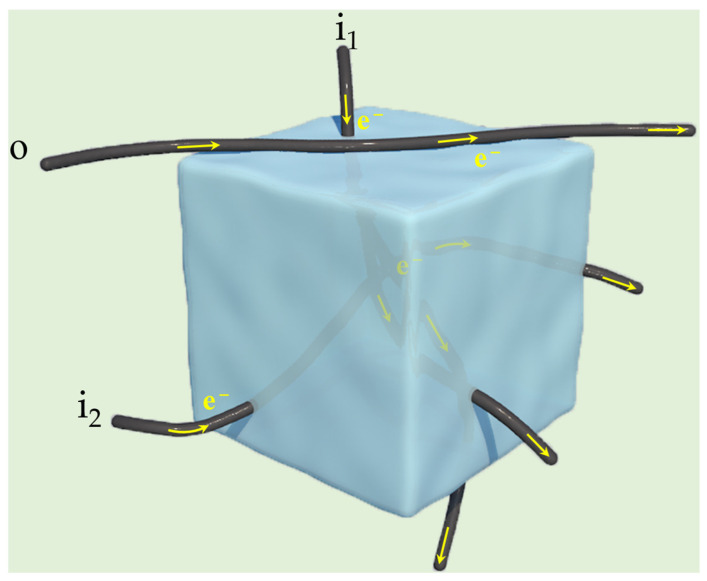
The schematic illustration shows research concept of this study. The coprecipitation method was employed to achieve the in situ growth of NaMnHCF on CNTs, where the CNTs in the NaMnHCF@CNT composite play the role of shortening the transport pathway of electrons and accelerating the electron motion.

In this work, we synthesized NaMnHCF composites in situ on CNTs through a facile coprecipitation method that produces no hazardous emissions. In the NaMnHCF/CNT composites, CNTs not only provide a fast diffusion channel for electrons but also shorten the transport pathway of electrons within the cathode electrode. The research concept for the synthesis of NaMnHCF is shown in Scheme 1. The obtained composites were studied in detail, including their phase structure, composition, and electrochemical properties. In addition, this study provides an interpretation of the improved cycling performance of NaMnHCF/CNTs. Specifically, the enhanced conductivity decreases the overpotential of the electrochemical reaction, thereby achieving better interfacial stability and a reduced corrosion rate. Furthermore, we also tried to uncover the main culprit responsible for the evolution from a two-plateau to single-plateau profile during the charge–discharge process of monoclinic NaMnHCF. This evolution is a common phenomenon observed by many researchers [24,25,26]. In our study, both the monoclinic phase (hydrated) and rhombohedral phase (anhydrated) could be identified when part of the two plateaus merged. Song et al. [26] revealed that the removal of crystal water could lead to the merging of the two plateaus (Fe^2+^/Fe^3+^ and Mn^2+^/Mn^3+^ redox) in the charge–discharge profile. This merging is a consequence of the coupled Fe^2+^/Fe^3+^ and Mn^2+^/Mn^3+^ redoxes, resulting from the balance of ionization energy and ligand field stabilization energy after water removal. The transformation of the monoclinic to the rhombohedral phase is an indicator that crystal water molecules are removed during cycling, which is likely to be responsible for the evolution of the charge–discharge profile based on the aforementioned analysis [27]. Finally, our study shows that compositing a certain mass of CNTs enhances the electronic conductivity of NaMnHCF and improves its cycling performance, implying that the conductive agent, when composited in situ, could offer a more positive effect than ex situ integration. As the coprecipitation method is the most suitable method for the large-scale production of low-cost NaMnHCF, this study is inspiring for the large-scale production of NaMnHCF with optimal electronic conductivity.

## 2. Results and Discussion

**Figure 1:**
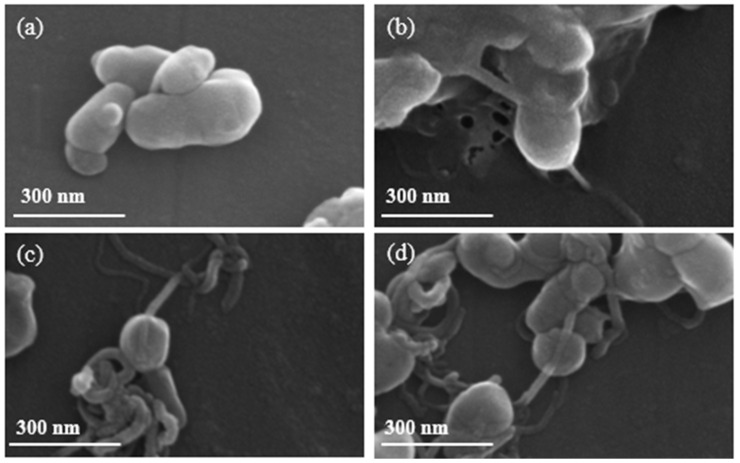
SEM images of (**a**) NaMnHCF, (**b**) NaMnHCF@2%CNT, (**c**) NaMnHCF@5%CNT, (**d**) NaMnHCF@10%CNT.

We first evaluated the in situ growth of NaMnHCF on CNTs with different CNT percentages. Figure 1 shows the formation of chain-like composite structures, where CNTs revert through NaMnHCF particles with different percentages of CNTs. The grain size of the composited and pristine samples ranges from 100 to 300 nm. In addition, the distribution of CNTs in the NaMnHCF@2%CNT and NaMnHCF@5%CNT samples is sparse and uneven (Figure S1b,c). In the NaMnHCF@10%CNT sample, the CNTs are either located on the surface or penetrating the bulk of NaMnHCF, with a higher content on the surface (Figure S1d,f). In contrast, the chain-like composite structures cannot be observed in the sample prepared using mechanical grinding only (Figure S1e), indicating that mechanical mixing is not effective for achieving intimate contact between CNTs and NaMnHCF.

**Figure 2:**
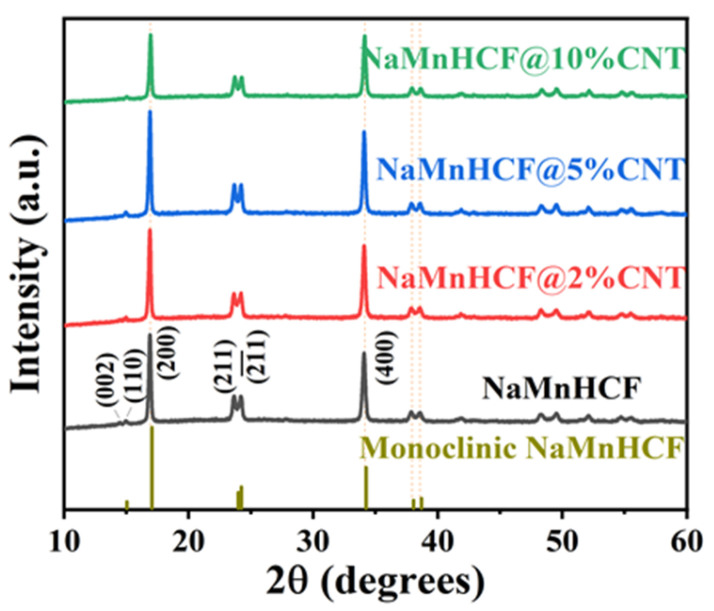
XRD patterns of NaMnHCF, NaMnHCF@2%CNT, NaMnHCF@5%CNT, and NaMnHCF@10%CNT.

The XRD measurements (Figure 2) reveal distinct peaks (002)/(110) and (211)/($\bar{2}\bar{1}1$) for both the pristine and composited samples, indicating the monoclinic structure of NaMnHCF. However, the absence of cleavage behavior in peak (400) implies that the NaMnHCF is not a perfect monoclinic structure, and some defects or Na^+^ vacancies are present in the structure. The results also show that the in situ composited CNTs have no effect on the crystal structure of the material.

**Figure 3:**
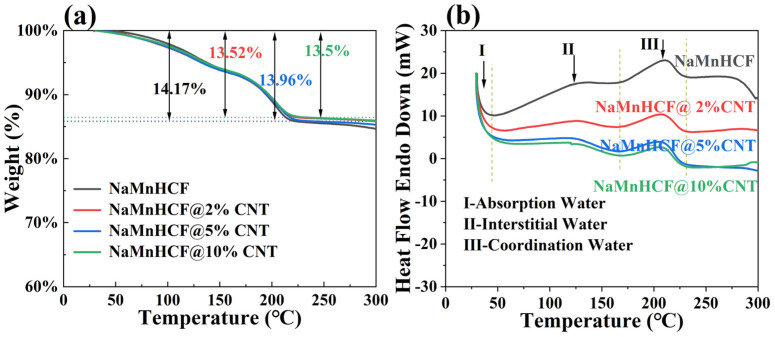
TG-DSC curves of NaMnHCF, NaMnHCF@2%CNT, NaMnHCF@5%CNT, and NaMnHCF@10%CNT: (**a**) TG, (**b**) DSC. The vertical dotted lines indicate the temperature ranges in which different type of water is removed in (**b**).

The TGA measurement results (Figure 3a) show that the water content of NaMnHCF, NaMnHCF@2%CNT, NaMnHCF@5%CNT, and NaMnHCF@10%CNT is 14.17%, 13.52%, 13.96%, and 13.5%, respectively. This shows that the in situ composition of CNTs during the synthesis of the NaMnHCF does not significantly reduce the water content of the material. Furthermore, as the temperature increases, the TGA plots of the mass percentage vs. temperature are nearly identical. This similarity suggests that the content of different types of water (absorbed water, interstitial water, and coordinated water) are similar in the material [1]. As can be seen from Figure 3b, the DSC curves of the four samples exhibit three heat flow peaks in the temperature range of 30–230 °C during the heating process. Peak I, II, and III represent the evaporation of adsorbed water, interstitial water, and coordinated water in the crystal structure, respectively [1]. For all the samples, the temperature ranges for the removal of the three types of water are consistent, indicating similar water-related defects within the NaMnHCF framework.

The chemical compositions of the samples were assessed using TGA and ICP-OES. The reaction conditions and the resulting chemical compositions of all the samples are listed in Table 1. Obviously, during the synthesis of the samples, the CNT content in the material increases proportionally with the amount of CNTs added to the reactor. This indicates that the in situ NaMnHCF/CNT composite can be achieved by the addition of CNTs during the synthesis process. When compared with the pristine sample, the Na content, [Fe(CN)_6_]^4−^ vacancy defects, and water content in the NaMnHCF/CNT composites exhibit only slight variations. These findings are in agreement with the XRD results.

**Figure 4:**
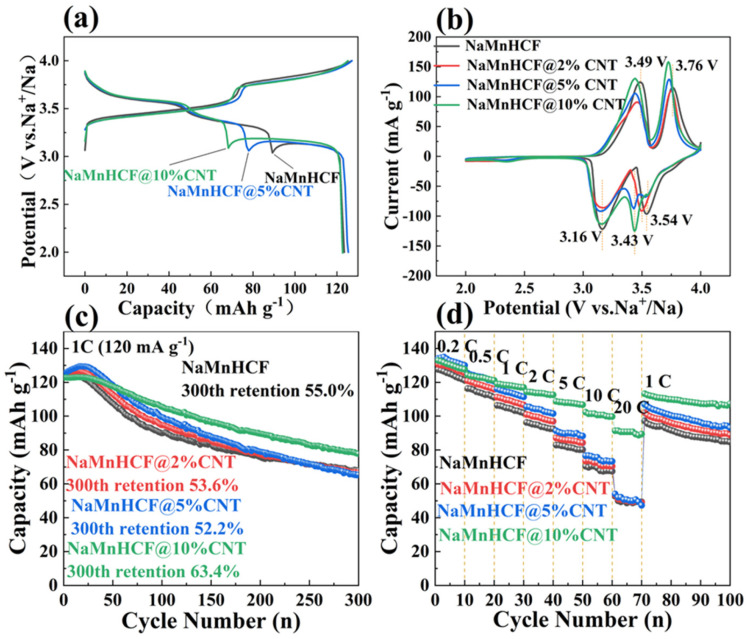
Electrochemical performance of NaMnHCF, NaMnHCF@2%CNTs, NaMnHCF@5%CNTs, and NaMnHCF@10%CNTs: (**a**) initial charge-discharge plots, (**b**) CV curves at 0.1 mV s^−1^ in the first cycle, (**c**) cycling performance at 1 C, and (**d**) rate capability.

In this study, our focus is on the effect of the composited CNTs on the electronic conductivity of NaMnHCF material. Generally, the in situ composite of CNTs is believed to promote electron mobility by providing a fast channel and shortening the transport pathway for electrons, thereby accelerating the charge–discharge rate. Figure 4 compares the electrochemical performance of NaMnHCF, NaMnHCF@2%CNT, NaMnHCF@5%CNT, and NaMnHCF@10%CNT. In the initial cycle, two distinct plateaus are observed, with the higher plateau representing the redox of Mn^3+/2+^ and the lower plateau the redox of Fe^3+/2+^, respectively [28]. To confirm the improved conductivity of NaMnHCF/CNT, we accessed the electrochemical performance of NaMnHCF/CNT with different CNT contents. Expectedly, the potential hysteresis decreases with the increasing CNT content (Figure 4a). Specifically, the in situ composite CNTs result in a lower voltage plateau for charge, a higher voltage plateau for discharge, and a reduction in the difference between the charge and discharge plateaus. The CV curves (Figure 4b) show that the oxidation potentials for Fe^3+/2+^ and Mn^3+/2+^ are situated at 3.49 V and 3.76 V, respectively. The oxidation potential of the composite samples decreases gradually with the increase in the CNT content, aligning with the results of the initial charge–discharge plots (Figure 4a). In the initial 10 cycles, NaMnHCF@2%CNT and NaMnHCF@5%CNT show an obvious activation process in the capacity delivery, whereas the capacity of NaMnHCF@10%CNT remains stable. After 300 cycles, the capacity retention of NaMnHCF, NaMnHCF@2%CNT, NaMnHCF@5%CNT, and NaMnHCF@10%CNT is 55.0%, 53.6%, 52.2%, and 63.4%, respectively (Figure 4c). Figure 4d demonstrates that the rate capability is particularly enhanced in NaMnHCF@10%CNT. It is conclusive that the composited CNTs improve both the cycling performance and rate capability of NaMnHCF. The NaMnHCF@10%CNT sample presents a specific capacity of 100 mA h g^−1^ and 90 mA h g^−1^ at a current density of 10 C and 20 C, respectively, outperforming the other samples which achieve ~70 mA h g^−1^ and 50 mA h g^−1^ at the same current densities. We believe that it is related to the sparse and uneven distribution of CNTs in the NaMnHCF@2%CNT and NaMnHCF@5%CNT samples; nevertheless, the CNTs are even and widely distributed in the NaMnHCF@10%CNT sample.

**Figure 5:**
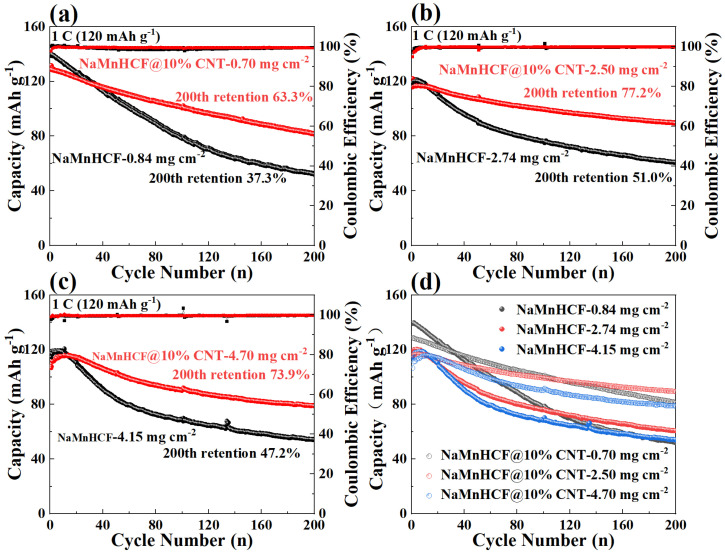
Cycling performance of NaMnHCF and NaMnHCF@10%CNT at various mass loadings: (**a**) low mass loading, (**b**) medium mass loading, (**c**) high mass loading, (**d**) comparison of the cycling performance at different mass loadings.

In order to deeply investigate the effects of in situ composited CNTs on the electrochemical performance of NaMnHCF, we evaluated the performance of NaMnHCF@10% CNT at different mass loadings. The optimal performance of NaMnHCF was observed at a mass loading of 2–3 mg cm^−2^, probably caused by the interfacial side reactions. We found that the cycling retention of pristine NaMnHCF is only 37.3% after 200 cycles at a low mass loading (~1 mg cm^−2^), while the NaMnHCF@10%CNT shows a capacity retention of 63.3% at the same mass loading (Figure 5a). At a higher mass loading of 2.5 mg cm^−2^, the capacity retention of NaMnHCF and NaMnHCF@10%CNTs after 200 cycles increased to 51.0% and 77.2%, respectively (Figure 5b). However, at an even higher mass loading (~4 mg cm^−2^), the cycling performance of both samples decreased, with NaMnHCF@10%CNT showing a capacity retention of 73.9% (Figure 5c). Overall, the cycling performance of NaMnHCF@10%CNT is better than that of pristine NaMnHCF in various mass loadings. After 500 cycles, NaMnHCF@10%CNT achieves an even higher capacity retention than what NaMnHCF achieves after 200 cycles at the same level of mass loading (Figure S2), and all the samples demonstrate the best cycling performance at the mass loading of 2–3 mg cm^−2^, which will be discussed later (Figure 5d).

**Figure 6:**
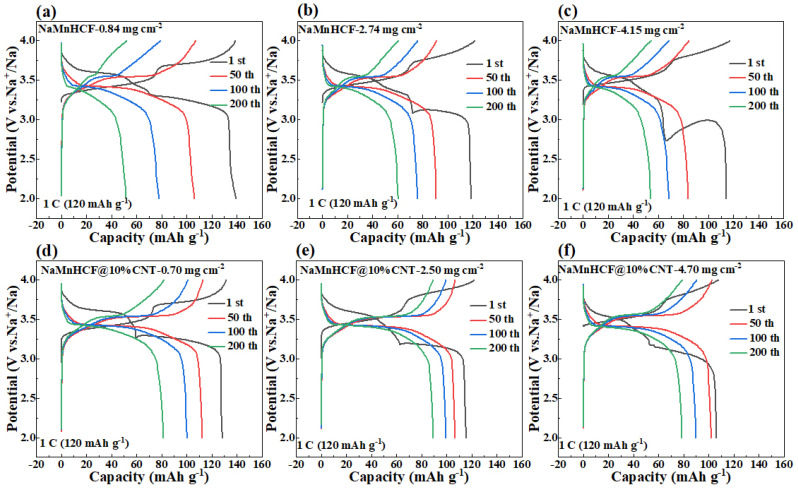
Charging and discharging plots of NaMnHCF and NaMnHCF@10%CNT electrodes at different mass loadings: (**a**,**d**) low mass loading, (**b**,**e**) medium mass loading, (**c**,**f**) high mass loading.

**Figure 7:**
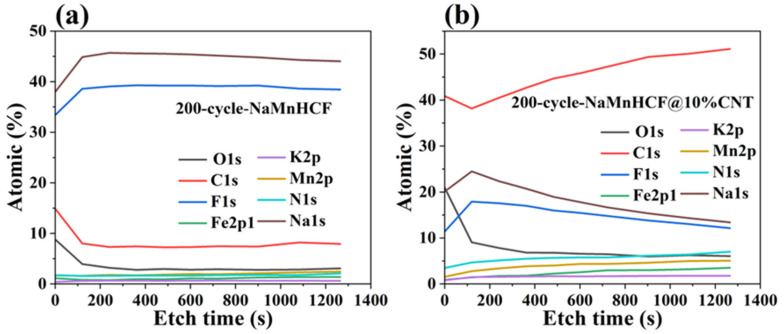
XPS depth profiling of cycled materials: (**a**) 200-cycle-NaMnHCF, (**b**) 200-cycle-NaMnHCF@10%CNT.

It is noteworthy to observe the dramatic difference in the cycling performance between NaMnHCF/CNT and NaMnHCF. Generally, enhancing the electronic conductivity boosts the rate capability rather than the structural stability or cycling performance. To explore the factors contributing to the cycling improvement, we made a detailed analysis of the charge and discharge plots for electrodes at different mass loadings (Figure 6). We found that the plateau in the charging process of both the NaMnHCF and NaMnHCF@10%CNT samples rises with the increase in mass loading, which is consistent with the CV results (Figure S3) in terms of polarization. In the first charge–discharge plot, there is a significant increase in potential polarization with increased mass loading for NaMnHCF. However, the increase in polarization is less obvious with higher mass loading for NaMnHCF@10%CNT. The XPS etching results for the two pristine samples (Figure S4) show that the contents of the Na and O elements decrease, while the content of the N, Mn, and Fe elements increase with the etching depth because of the CMC layer on the particles, with the elemental content stabilizing after approximately 500 s of etching. After 200 cycles, the XPS and XPS etching results of the two samples are quite different; the XPS spectrometry of F1s shows that the 200-cycle-NaMnHCF sample contains a higher content of the F element than the 200-cycle-NaMnHCF@10%CNT (Figure 7 and Figure S5). The F element predominates on the surface of the 200-cycle-NaMnHCF, comprising up to 35% of its elemental content. In contrast, the F content is around 20% on the surface of 200-cycle NaMnHCF@10%CNT. The dramatic difference in the F element concentration implies different side reaction rates as the F element originates from FEC in the electrolyte. The DFT and experiment have implied the side reactions related to FEC [29,30]. Specifically, FEC firstly decomposes to CO_2_ and 2-fluoroacetaldehyde radical cation by transferring one electron to the cathode electrode, and then, the 2-fluoroacetaldehyde radical cation transforms to the acetaldehyde radical cation and F^−^ by transferring one more electron to the electrode. In the end, the radical center of the acetaldehyde radical cation attacks the carbonyl carbon of FEC to form aldehyde and oligomers of alkyl carbonates. The side reactions on the NaMnHCF@10%CNT/electrolyte interface are obviously suppressed when compared to NaMnHCF. This reduction facilitates the interfacial stability of NaMnHCF@10%CNT and its cycling performance (Figure 7a,b). The side reactions are highly related to the surface potential of the cathode. The surface potential of an electrode material is defined as the work needed to move an electron to the electrode surface from the remote distance in the vacuum. The surface potential indicates the energy of a charged particle (electron or ion). According to electrostatic theory, the higher the potential, the lower energy the electron. The oxidation reactions of an electrolyte always occur on the surface of high potential (low energy for electrons) because the electrons tend to move from electrolyte molecules (a high-energy level) to the surface of the cathode (a low-energy level). Figure 6 indicates that our strategy improves the electronic conductivity so that the required voltage to move the electron from the cathode to the anode is decreased, meaning that the surface potential on the cathode can be decreased, and the electron energy can be lifted on the cathode, suppressing the oxidation reactions of electrolyte and side reactions.

The in situ composited CNTs can accelerate electron mobility, thereby shortening the electron transport pathway. This reduction in the pathway decreases the overpotential during the charge–discharge process. Consequently, the resultant lower polarization leads to reduced potential at the surface of the cathode material. Therefore, the side reactions between the cathode material and electrolyte are suppressed, which, in return, improves the cycling performance of the material.

In the charge–discharge curves of NaMnHCF, the two potential plateaus constantly evolved into a single medium-potential plateau in the cycling process. This phenomenon is common in our data (Figure 6) and corroborated by the published literature [28]. Typically, hydrated monoclinic NaMnHCF presents two plateaus in the charge–discharge plot, with a higher plateau attributed to Mn^3+/2+^ and a lower one to Fe^3+/2+^. Meanwhile, it has been reported that the two potential plateaus of NaMnHCF could merge into a single plateau because of the removal of crystal water from the NaMnHCF framework due to the phase transition from the monoclinic phase (M-phase, hydrated) to the rhombohedral phase (R-phase, anhydrated) [28]. To elucidate this evolution of the potential plateaus, Wu et al. carried out a deep investigation of the changes in crystal structure and electronic structure after erasing crystal water from the theoretical perspectives [28]. They provided an interpretation that the removal of crystal water couples the redox reaction of Fe^3+/2+^ and Mn^3+/2+^, resulting in the transition from a two-plateau to a single-plateau plot. Our study reveals that the plateau evolution is gradual rather than abrupt [28]. While two distinct plateaus remain in the charge–discharge plot after 10 cycles, they merged into a single plateau after 15 cycles, and the new plateau is located between the higher and lower plateau of the initial cycle. The two plateaus completely merge after 20 cycles (Figure S6).

**Figure 8:**
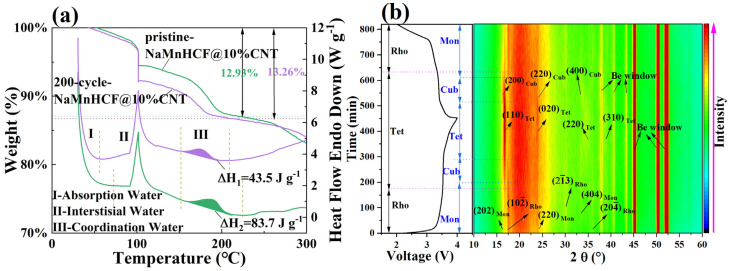
(**a**) TG-DSC of pristine and 200-cycle-NaMnHCF@10%CNT, (**b**) 2D contour plots of the in situ XRD pattern of NaMnHCF@10%CNT from 10° to 60° of 2θ, showing the structural evolution of the cathode material in the 13th cycle. The vertical dotted lines indicate the temperature ranges in which different type of water is removed in (**a**). The horizontal dotted lines indicate the points at which the phase transition takes place of monoclinic (Dark Blue) and Rhombohedral (magenta) in (**b**).

To investigate the evolution of this plateau, we employed experimental methods that have not been previously reported. We carried out the TG-DSC on both pristine and 200-cycle NaMnHCF@10%CNTs (Figure 8a). The testing procedures involved gradually increasing the temperature from 30 °C to 100 °C at a rate of 10° min^−1^, followed by a 30 min hold to remove adsorbed water from the material. Subsequently, the temperature was increased from 100 °C to 600 °C at a rate of 10 °C min^−1^. Notably, the 200-cycle-NaMnHCF@10%CNT sample exhibits greater weight loss at 100 °C, which is due to the adsorption of ambient moisture during the transfer of the sample from the glove box to the TGA instrument. Both materials displayed a heat absorption peak between 150 °C and 220 °C, which is attributed to the evaporation of the coordinated water [31], and it is clear that the material has a lower coordinated water content and less weight loss after cycling. The decreased coordinated water content suggests that the material experiences gradual dehydration during the cycling process. To support this, we performed in situ XRD tests of NaMnHCF@10%CNT (Figure 8b). Normally, there is only a structural evolution, which is from the M-phase to the C-phase (Cubic phase) to the T-phase and back to the C-phase and back to the M-phase in the M-phase material’s charge and discharge (Figure S7a); however, in the 13th cycle, both the M-phase and R-phase of NaMnHCF were initially present, indicating that part of the M-phase is transformed into the R-phase as the cycle proceeds. It is also observed that the two distinct plateaus become vague, accompanied by a freshly formed plateau in the charge–discharge plot (Figure S7b) [32]. Both the M-phase and R-phase NaMnHCF can be identified in the in situ XRD patterns. In the in situ X-ray diffraction (XRD) patterns, the peaks at 16.3° and 30° were assigned to (202)_M-phase_ and (2$\bar{1}$3)_R-phase_, respectively. This indicates a partial transformation from the M-phase to the R-phase in the 13th cycle. Interestingly, the peak at 16.9°, probably assigned to the tetrahedral phase (T-phase), is existent in the initial charge state, probably indicating an incomplete discharge. As charging proceeds, the M-phase evolves to the C-phase, indicated by the weakening and disappearance of the (202)_M-phase_ peak at 16.3°. The C-phase continues evolving and, ultimately, reaches the T-phase. Simultaneously, the R-phase transforms to the T-phase at the end of charge, indicated by the upshift and weakening intensity of the (2$\bar{1}$3)_R-phase_ peak at 30°. As the R-phase material contains no crystal water, we can, therefore, conclude that the T-phase originating from the R-phase also contains no water. In addition, we found that both the M-phase and R-phase evolve into the same T-phase by the end of the charge. To validate and confirm whether the M-phase (hydrated) and R-phase (anhydrated) NaMnHCF terminate in the same T-phase, we compared our results with those reported by Goodenough et al. [26]. The XRD pattern of fully charged monoclinic NaMnHCF (Figure S8a) is identical to that of fully charged rhombohedral NaMnHCF (Figure S8b). The aforementioned evidence implies that the T-phase is likely to be free of water, and the water molecules are removed in the charge process of M-phase NaMnHCF. Based on our experiments, coupled with the evidence reported by others, we may speculate that the convergence of two plateaus observed in the charge–discharge process may be partially due to the loss of crystal water molecules.

To confirm that the enhancement in electrochemical performance is attributable to the improved electronic conductivity, a four-point probe was conducted to measure the resistivity of the cathode electrode. The specific resistance of NaMnHCF and NaMnHCF@10%CNT is 4.675 kΩ cm and 2.783 kΩ cm at the mass loading of 3.25 mg cm^−2^ (Table 2). When the mass loading is increased to 4.32 mg cm^−2^, the specific resistance rises to 4.979 and 3.15 kΩ·cm, respectively, for NaMnHCF and NaMnHCF@10%CNT. In both cases, the resistance of NaMnHCF@10%CNT is reduced by about 40% compared with that of NaMnHCF. This suggests that the introduction of CNTs greatly enhances the electronic conductivity of NaMnHCF@10%CNT.

Furthermore, we conducted the electrochemical impedance spectroscopy (EIS) test (Figure S9), and the fitting results are presented in Table S1. We found that the difference in ohmic resistance (Rs) between NaMnHCF@10%CNT and NaMnHCF is minimal. The difference in the interfacial resistance (Rf) and charge transfer resistance (Rct) between NaMnHCF@10%CNTs and NaMnHCF is irregular. This indicates that the introduction of CNTs does not affect the Rs, Rf, and Rct of NaMnHCF to a detectable degree using EIS. In addition, we performed a galvanostatic intermittent titration test (GITT) (Figure S10), which shows that the diffusion coefficients of the materials before and after CNT composition are approximately equal.

## 3. Experimental Section

Synthesis of materials: Pristine NaMnHCF was prepared as follows: 0.015 mol of Na_4_Fe(CN)_6_·10H_2_O, 0.3 mol of NaCl, 0.2 g of PVP, and 0.03 mol of MnSO_4_ were weighed, respectively. PVP was dissolved into 100 mL of deionized water and stirred constantly using a magnet stirrer. Once the PVP was completely dissolved, Na_4_Fe(CN)_6_·10H_2_O and NaCl were added. A pale-yellow solution (A) was obtained after Na_4_Fe(CN)_6_·10H_2_O and NaCl were dissolved. MnSO_4_ was dissolved into 50 mL of deionized water and stirred using a magnet until a light pink solution (B) was formed. Then, Solution B was gradually added into Solution A at a rate of 1.0 mL min^−1^, using peristaltic pump under stirring condition at 25 °C. After 50 min, Solution B was completely added into Solution A, and the reaction was aged for 23 h at a constant temperature and stirring rate. Once the reaction was completed, the suspension was filtered and washed with deionized water three times to remove the impurity ions. Finally, the separated precipitate was dried in a vacuum oven at 80 °C for 12 h to obtain white NaMnHCF powder.

To synthesize NaMnHCF containing 2 wt%, 5 wt%, or 10 wt% CNTs, 1.9 g, 4.9 g, or 10.4 g of 5 wt% CNTs dispersion were added to Solution A, respectively. These samples were designated as NaMnHCF@2%CNT, NaMnHCF@5%CNT, and NaMnHCF@10%CNT, respectively.

Material characterizations: The morphology of the samples was observed using a scanning electron microscope (FESEM, Hitachi S3400N). The crystal structure of the samples was analyzed using an X-ray diffraction (XRD, Rigaku MiniFlex 600) instrument. The XRD measurements were performed at a scanning rate of 10° min^−1^, with Cu Kα radiation (λ = 1.5406 Å) at an applied voltage of 40 kV and a current of 15 mA. We detected the water content using a thermogravimetric analyzer with ramp rate of 10 °C min^−1^ (TGA, NETZSCH STA449C) under a nitrogen flow of 20 mL min^−1^. The molar ratio of Na, Fe, and Mn was determined using the an inductively coupled plasma optical emission spectrometry (ICP-OES, the Perkin Elmer Avio 500) instrument. We performed X-ray photoelectron spectroscopy (XPS) using an ESCALAB Xi+ spectrometer.

XPS depth profiling: X-ray photoelectron spectroscopy (XPS) was performed on an ESCALAB Xi+ spectrometer equipped with a monochromated Al target. XPS survey was conducted with a pass energy of 160 eV and an energy step size of 1.0 eV. The spectrum of different elements was acquisited with a pass energy of 40 eV and an energy step size of 0.1 eV, in which the spot diameter of the X-ray beam is 650 μm. Depth profiling was completed by employing monatomic EX06 ion source in which sputtering occurs every 30 s. The content of elements was obtained by analyzing the peak areas of the element photoelectron spectrum. The peak position was calibrated based on the 285.0 eV of C-C bond.

In situ XRD measurements [33]: We conducted in situ XRD using a device specially designed for XRD measurement using Rigaku MiniFlex 600, which was purchased from Beijing Scistar Technology Co., Ltd. (Beijing, China) Previous reports interpreted the structure of the device [34,35,36]. A free-standing electrode for in situ cell was prepared by pressing the active material into a pellet, aiming to increase the peak intensity of the XRD pattern of the active material and eliminate peaks originating from the current collector. Specifically, the active material (70 wt%) and carbon nanotubes (30 wt%) were mixed using a mortar and pestle for a duration of 20 min. Subsequently, the active material and carbon nanotubes were subjected to a pressure of 10 MPa for 1 min, resulting in the formation of cathode pellets. These pellets could be utilized as cathode electrodes for in situ cell. First, the cathode pellet was carefully positioned in direct contact with the Beryllium window (Be Window), followed by adding the separator (glass fiber), electrolyte (1.0 M NaPF_6_ in PC:FEC = 97:3 vol %), and sodium metal in order. Finally, after sealing it with plastic rings and screws, we completed assembling the in situ cell. The structural evolution was revealed by recording the XRD pattern of the cathode material every 15 min during the in situ XRD measurement in a range of 10–60°, with a step size of 0.02° at a rate of 10° min^−1^. The in situ cell was subjected to a charging rate of 0.2 C, followed by a discharging rate of 0.2 C (1 C = 120 mA g^−1^).

Four-Probe Method for Determining Resistivity: The test conditions required a block or film sample with a diameter greater than 1cm and a thickness greater than 1mm. The four probes 1, 2, 3, and 4 were arranged in a straight line and equally spaced apart. The current loop (*I*) was emitted by Probe 1 and collected by Probe 4, while Probes 2 and 3 formed a voltage loop (ΔV). Afterwards, the specific resistance of the electrode can be obtained by applying the formula:

$$\rho =\frac{\pi }{\ln\;2}\;\frac{\Delta V}{I}=4.53236\;\frac{\Delta V}{I}.$$

Cell assembly and electrochemical tests: The cathode material, carbon black (Super P), carbon nanotubes (CNTs), and carboxy-methyl cellulose (CMC) were ground together with a mortar and pestle for 15 min at a weight ratio according to the content of CNTs in various samples, with the ratio of NaMnHCF:Super P:CNT:CMC set to 7:1:1:1. Cathode slurry was prepared by adding an appropriate amount of distilled water, followed by grinding for an additional 10 min. This as-prepared slurry was then coated onto a piece of carbon-coated Al foil, and the resulting electrode was dried in a vacuum oven at 80 °C for 12 h. With a mass loading of approximately 1.5 mg cm^−2^, the cathode electrode was further punched into a 10 mm disk shape. This disk was subsequently utilized as the cathode electrode in coin cells. The 2032-type coin cells were fabricated in a glove box filled with Argon, where the level of H_2_O and O_2_ was maintained below 0.1 ppm. The cells consisted of an electrolyte containing 1.0 M NaClO_4_ dissolved in a mixture of propylene carbonate (PC) and fluoroethylene carbonate (FEC) in a ratio of 90:10 (*v*/*v*). The anode utilized in these cells was Na metal. To assess the cycling stability and rate capability of the cathode materials, galvanostatic charge–discharge tests were conducted using a Neware CT-4000 workstation. These tests were performed within a voltage range of 2.0 to 4.0 V. Cyclic voltammetry (CV) was measured using a CHI 760C (Chenhua Instruments Ins. Shanghai, China) electrochemical workstation. Furthermore, electrochemical impedance spectroscopy (EIS) was carried out using a CHI 760C (Chenhua Instruments Ins. Shanghai, China) electrochemical workstation. The frequency range for the EIS measurements was set from 0.1 Hz to 100,000 Hz, with an applied amplitude of 5 mV.

## 4. Conclusions

In this work, we conducted a systematic investigation into the effects of the in situ introduction of CNTs on the electrochemical performance of NaMnHCF, as well as the mechanism behind these effects. A small amount of CNTs added to NaMnHCF was not sufficient to increase the electronic conductivity. However, increasing the percentage of CNTs results in deeper penetration into the NaMnHCF particle bulk, shortening the transport pathway of electrons and accelerating the electron motion. This enabled the specific capacity to be increased from 50 to 90 mA h g^−1^ at a current density of 20 C. The in situ growth of NaMnHCF on CNTs also enhances the cycling stability of NaMnHCF. This improvement is attributed to reduced polarization from the introduction of CNTs, which, in turn, reduces the side reactions in the NaMnHCF/electrolyte interface. Moreover, we discovered the loss of crystal water during the charge–discharge process, which probably results in the evolution from the M-phase two-plateau to the R-phase one-plateau profile during the cycling process. These results provide valuable insights into producing NaMnHCF with high electronic conductivity and a deeper understanding of the behavior of crystal water during the charge–discharge process. Such insights are expected to facilitate the practical application of PBAs not only in the sodium-ion batteries but also in other alkaline-ion batteries.

## Figures and Tables

**Table 1:** Synthesized sample conditions and the chemical composition of the samples calculated from the data of ICP and TGA.

Denotation	Synthesized Sample Conditions		Chemical Composition of Sample	Actual CNT Content
	Na^+^:Fe(CN)_6_^4−^:Mn^2+^	5 wt% CNT Dispersion Added Mass		
NaMnHCF	36:1.5:3	0	Na_1.51_Mn[Fe(CN)_6_]_0.87_·2.70H_2_O	0
NaMnHCF @2%CNT	36:1.5:3	1.9 g	Na_1.44_Mn[Fe(CN)_6_]_0.86_·2.55H_2_O	1.1%
NaMnHCF @5%CNT	36:1.5:3	4.9 g	Na_1.42_Mn[Fe(CN)_6_]_0.86_·2.74H_2_O	4.2%
NaMnHCF @10%CNT	36:1.5:3	10.4 g	Na_1.56_Mn[Fe(CN)_6_]_0.87_·2.74H_2_O	5.7%

**Table 2:** Four-electrode impedance results for electrode sheets.

Denotation	Test Point	Resistance Value (kΩ cm)	Average Resistance Value (kΩ cm)
NaMnHCF—4.32 mg cm^−2^	1	5.75	4.979
	2	4.9375	
	3	4.25	
NaMnHCF—3.25 mg cm^−2^	1	5	4.675
	2	4.65	
	3	4.375	
NaMnHCF @10%CNT—4.32 mg cm^−2^	1	3.525	3.15
	2	2.8125	
	3	3.1125	
NaMnHCF @10%CNT—3.25 mg cm^−2^	1	2.8375	2. 783
	2	2.6875	
	3	2.825	

## Data Availability

Data are contained within the article and supplementary materials.

## Supplementary Materials

The following supporting information can be downloaded at: https://www.mdpi.com/article/10.3390/molecules29020313/s1. Figure S1. SEM images of (a) NaMnHCF, (b) NaMnHCF@2%CNT, (c) NaMnHCF@5%CNT, (d,f) NaMnHCF@10%CNT(e) the sample NaMnHCF/CNT composite prepared by mechanical grinding. Figure S2. Cycling performance of NaMnHCF@10%CNT electrode at different mass loadings. Figure S3. CV curves at 0.1 mV s^−1^ in the first cycle: (a) NaMnHCF, (b) NaMnHCF@10%CNT. Figure S4. XPS depth profiling of pristine materials: (a) NaMnHCF, (b) NaMnHCF@10%CNT. Figure S5.XPS spectrometry of F1s of 200-cycle materials. Figure S6. Charging and discharging plots of NaMnHCF@10% electrode in the different cycle. Figure S7. (a) The enlargement of in-situ XRD pattern in a narrow 2θ range of 15°–19°, 21°–25°, 28°–32°, showing the phases present during the structural evolution of the normal monoclinic crystal materials in the 1th cycle, (b) The enlargement of in situ XRD pattern in a narrow 2θ range of 15°–19°, 21°–25°, 28°–32°, the time interval of each pattern is 90 mins, showing the coexistence of monoclinic and rhombohedral phases and their structural evolution in the 13th cycle. Figure S8. (a) the XRD pattern of fully charged monoclinic NaMnHCF, (b) the in situ XRD pattern of rhombohedral NaMnHCF reported by Goodenough et al. Copyright 2015 American Chemical Society. Figure S9. EIS spectra of the samples: (a) NaMnHCF low mass loading, (b) NaMnHCF medium mass loading, (c) NaMnHCF high mass loading, (d) NaMnHCF@10%CNT low mass loading, (e) NaMnHCF@10%CNT medium mass loading, (f) NaMnHCF@10%CNT high mass loading. Table S1. Results of EIS fitting. Figure S10. GITT curves of the half cells with different samples as cathode materials: (a) NaMnHCF different mass loadings, (b) NaMnHCF@10%CNT different mass loadings, (c) NaMnHCF and NaMnHCF@10%CNT different mass loadings and (d) Logarithm of the chemical diffusion coefficient of Na^+^ as a function of stoichiometry calculated from GITT.
