# Supplementary material for: In Situ Growth of Sodium Manganese Hexacyanoferrate on Carbon Nanotubes for High-Performance Sodium-Ion Batteries
Source: Molecules. 2024 Jan 8;29(2):313. doi: 10.3390/molecules29020313 (PMC10821419; doi:10.3390/molecules29020313)
Supplement: Supplementary file 1 [file molecules-29-00313-s001.zip › molecules-2762119-supplementary.pdf]

Supporting information

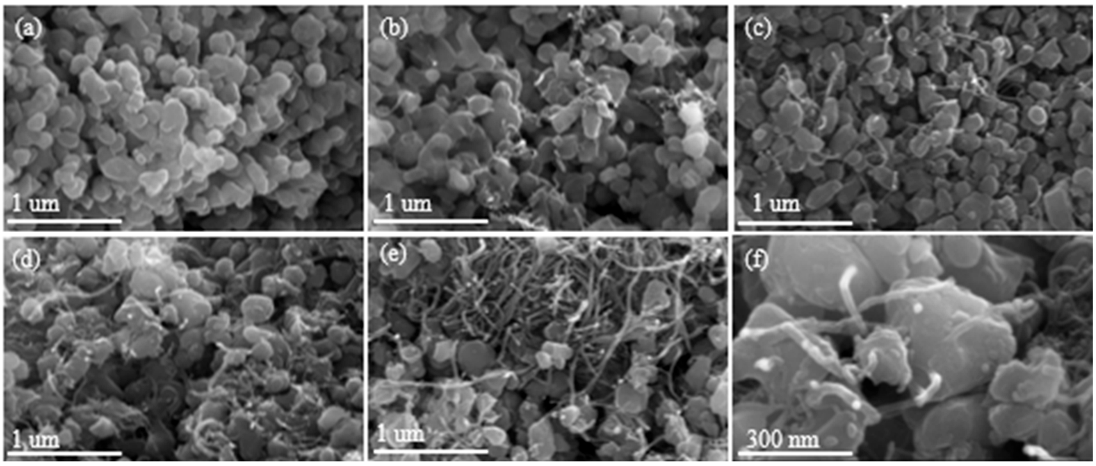

Figure S1. SEM images of (**a**) NaMnHCF, (**b**) NaMnHCF@2%CNT, (**c**) NaMnHCF@5%CNT, (**d**,**f**) NaMnHCF@10%CNT, (**e**) the sample NaMnHCF/CNT composite prepared by mechanical grinding.

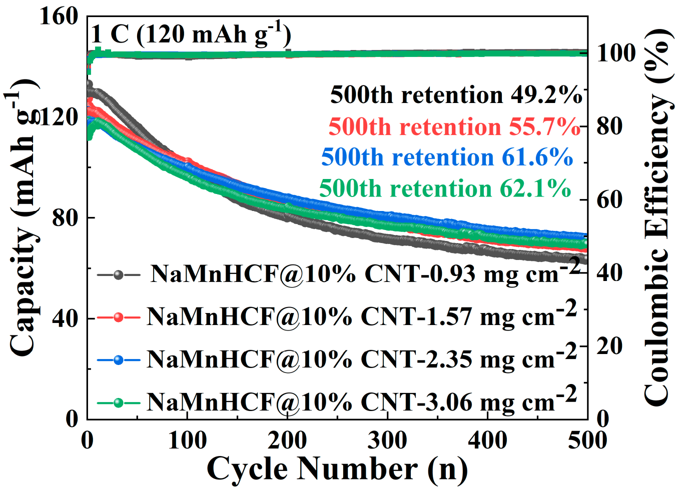

Figure S2. Cycling performance of NaMnHCF@10%CNT electrode at different mass loadings.

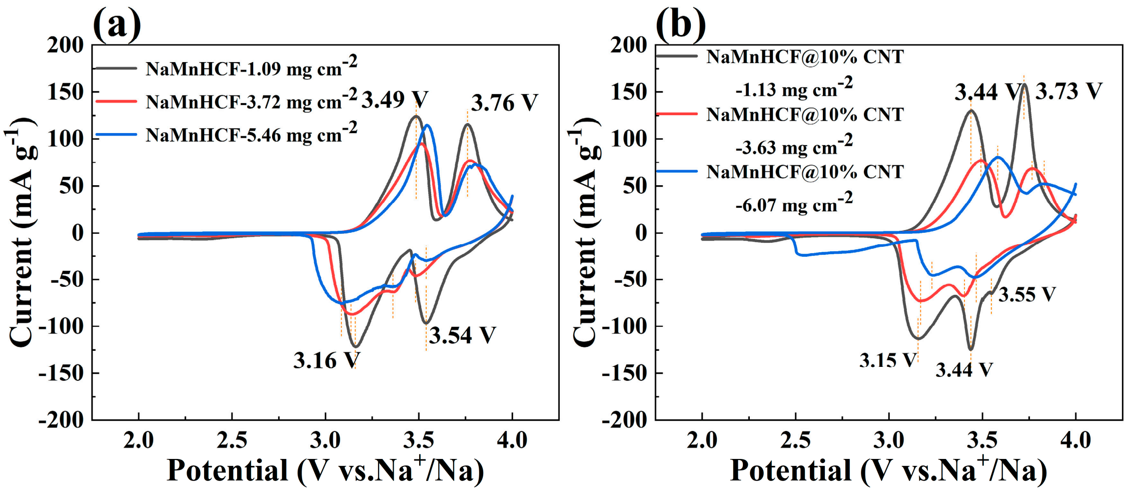

Figure S3. CV curves at 0.1 mV s^{-1} in the first cycle: (**a**) NaMnHCF, (**b**) NaMnHCF@10%CNT.

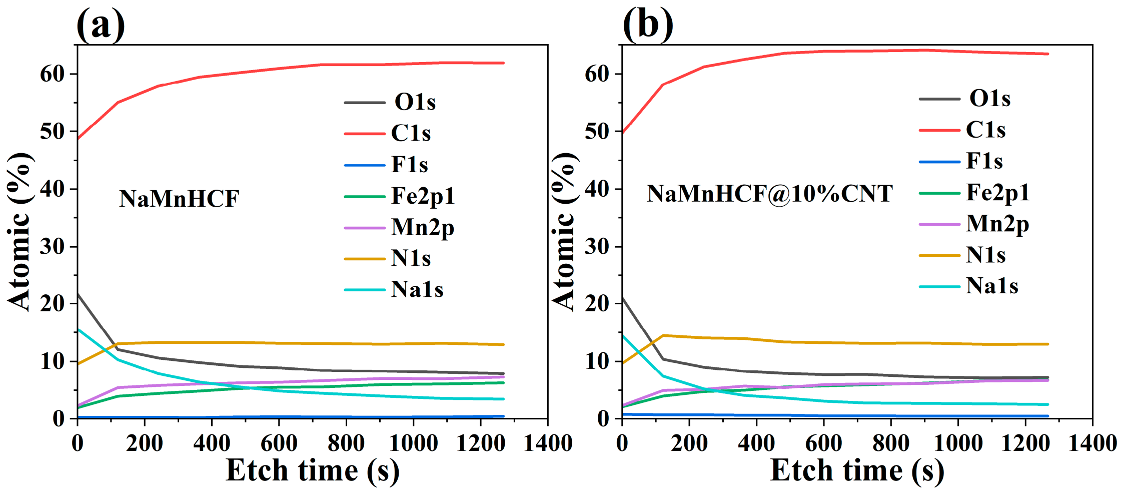

Figure S4. XPS depth profiling of pristine materials: (**a**) NaMnHCF, (**b**) NaMnHCF@10%CNT.

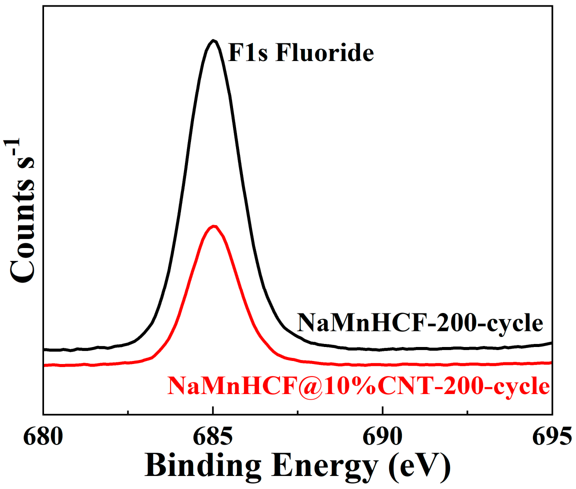

Figure S5. XPS spectrometry of F1s of 200-cycle materials.

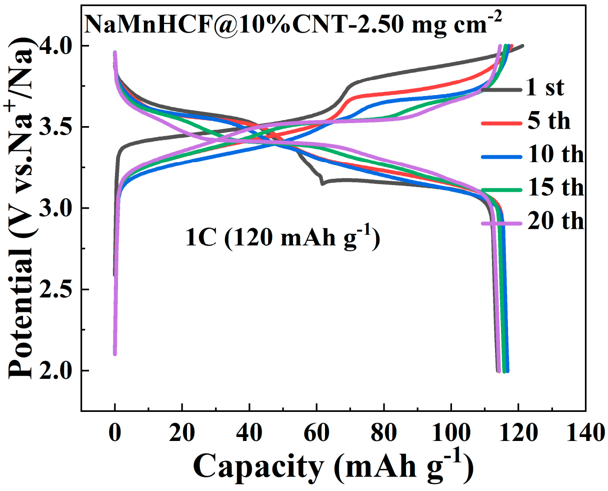

Figure S6. Charging and discharging plots of NaMnHCF@10% electrode in the different cycle.

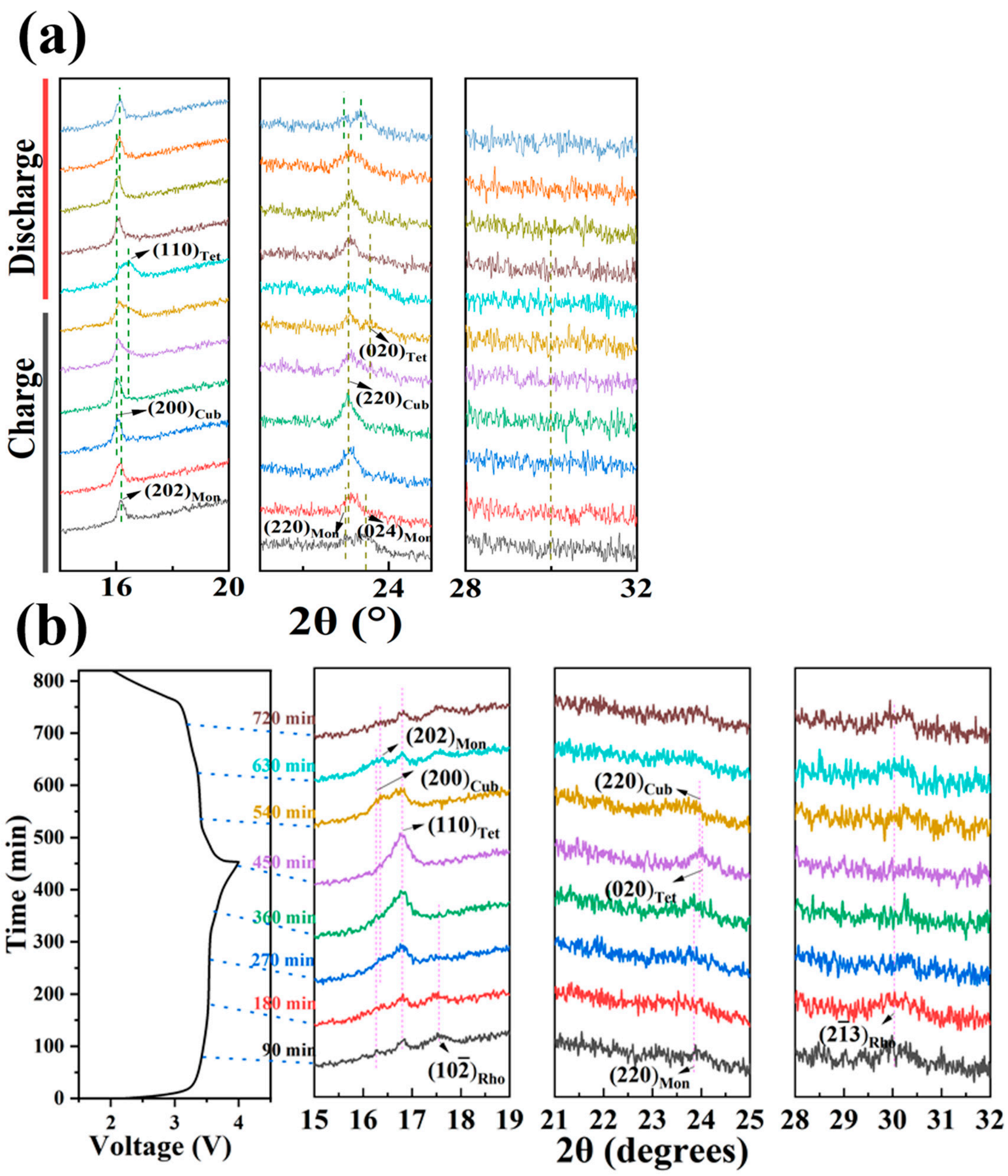

Figure S7. (**a**) The enlargement of in situ XRD pattern in a narrow 2θ range of 15°–19 °, 21°–25°, 28°–32°, showing the phases present during the structural evolution of the normal monoclinic crystal materials in the 1th cycle, (**b**) The enlargement of in situ XRD pattern in a narrow 2θ range of 15°–19°, 21°–25°, 28°–32°, the time interval of each pattern is 90 mins, showing the coexistence of monoclinic and rhombohedral phases and their structural evolution in the 13th cycle.

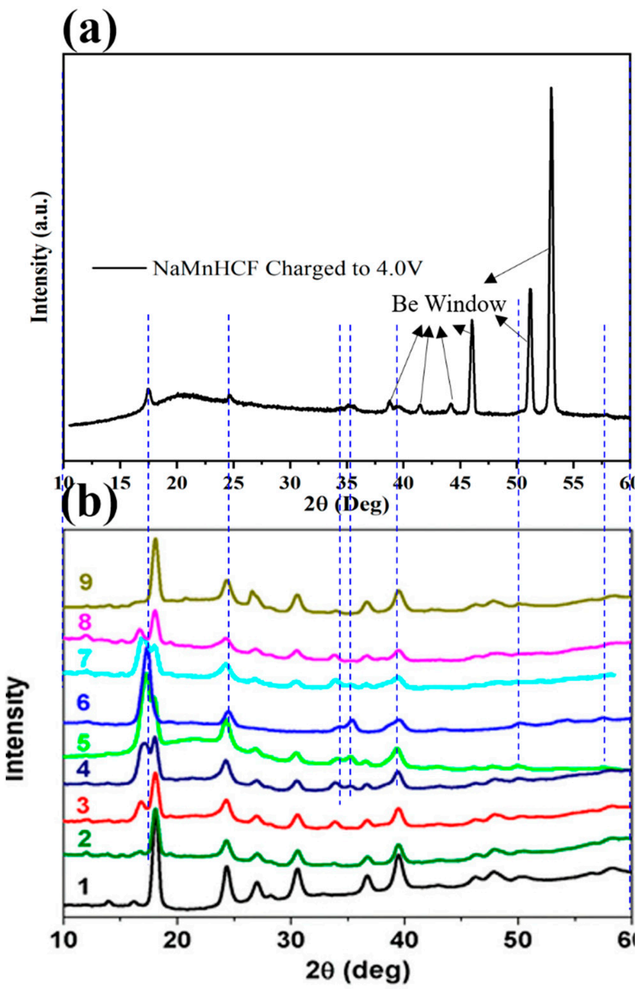

Figure S8. (**a**) the XRD pattern of fully charged monoclinic NaMnHCF, (**b**) the in situ XRD pattern of rhombohedral NaMnHCF reported by Goodenough et al. [1] Copyright 2015 American Chemical Society.

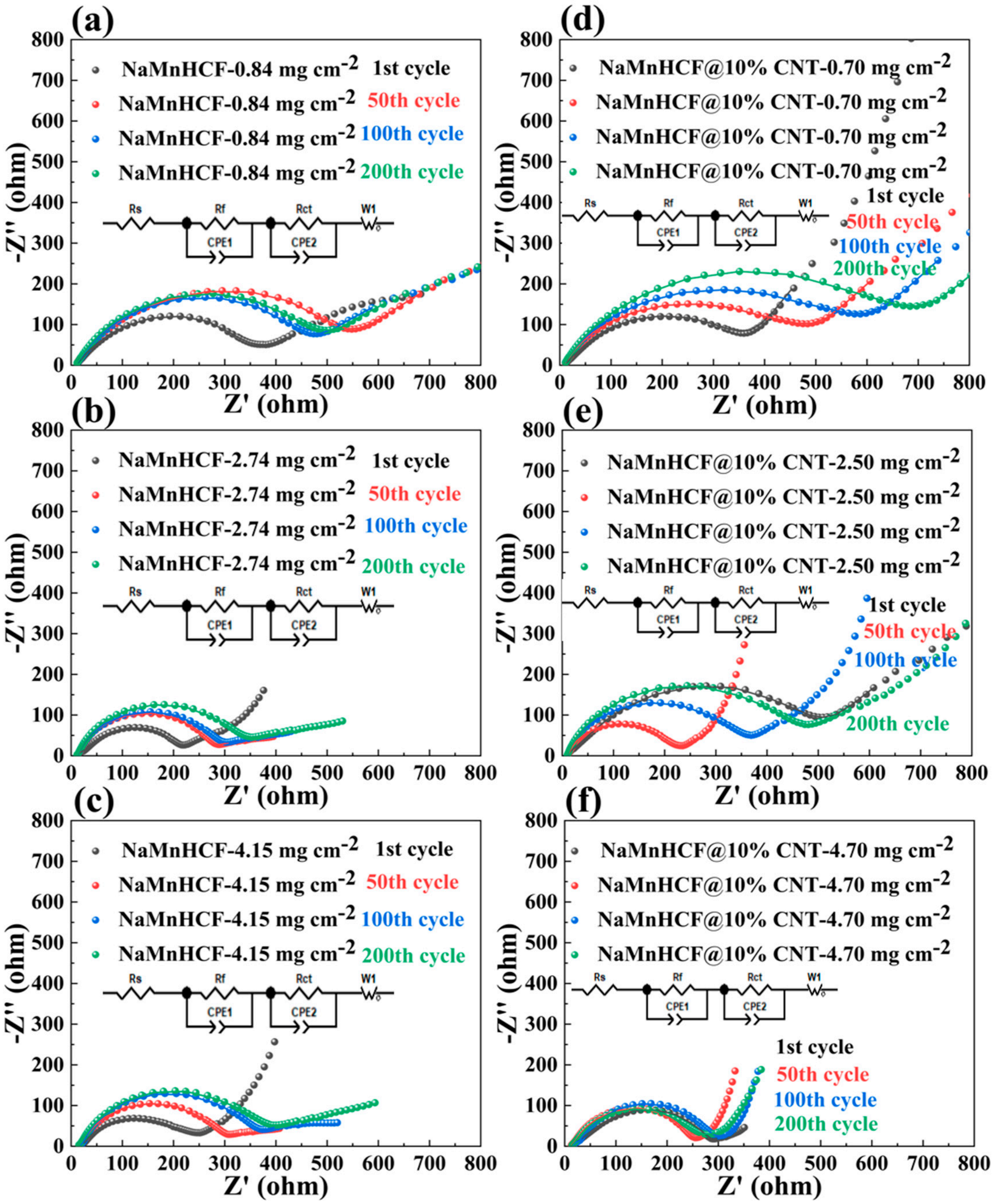

Figure S9. EIS spectra of the samples: (**a**) NaMnHCF low mass loading, (**b**) NaMnHCF medium mass loading, (**c**) NaMnHCF high mass loading, (**d**) NaMnHCF@10%CNT low mass loading, (**e**) NaMnHCF@10%CNT medium mass loading, (**f**) NaMnHCF@10%CNT high mass loading.

Table S1. Results of EIS fitting.

Denotation	Cycle	R_s	R_f	R_{ct}	R_{ct}	R_f	R_s	Cycle	Denotation
NaMnHCF—0.84 mg cm^{-2}	1st	8.3	118.7	192.1	10.3	340.3	10.2	1st	NaMnHCF @10%CNT—0.70 mg cm^{-2}
	50th	8.7	158.6	315.9	242.1	203.9	8.3	50th	
	100th	8.9	223.1	190.2	192.2	347.9	8.2	100th	
	200th	8.1	117.5	294.9	39.7	596	8.3	200th	
NaMnHCF—2.74 mg cm^{-2}	1st	10.1	180.2	17.59	55.6	411.3	8.9	1st	NaMnHCF @10%CNT—2.50 mg cm^{-2}
	50th	11.5	251.4	1.164	0.008	86.1	9.6	50th	
	100th	11.2	262.2	0.802	55.8	285	9.7	100th	
	200th	10.5	301	0.685	417	440.9	9.5	200th	
NaMnHCF—4.54 mg cm^{-2}	1st	15.8	179.6	3.3	71.2	212.8	4.1	1st	NaMnHCF @10%CNT—4.70 mg cm^{-2}
	50th	16.4	258.5	2.6	10.3	219.8	11.8	50th	
	100th	16.9	319	2.2	8.6	257.3	17.1	100th	
	200th	13.5	334.6	1.6	3.2	224.4	11.9	200th	

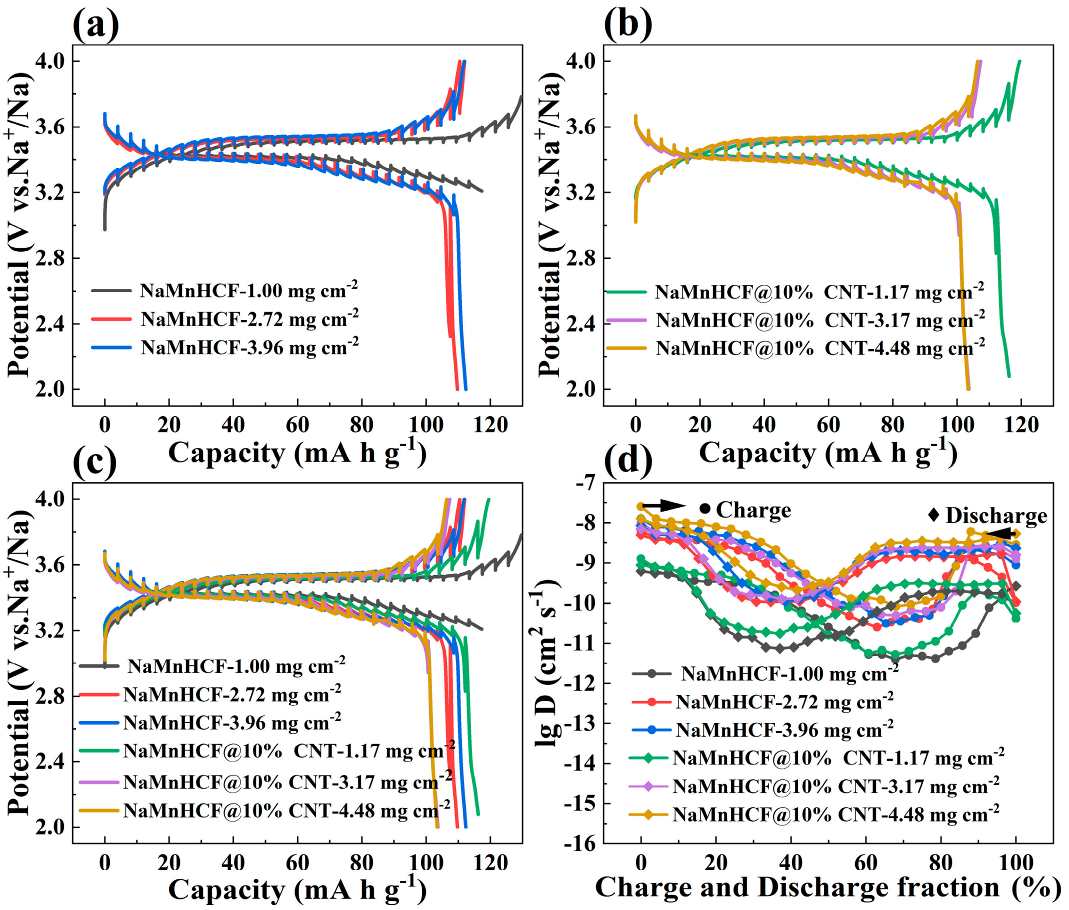

Figure S10. GITT curves of the half cells with different samples as cathode materials: (**a**) NaMnHCF different mass loadings, (**b**) NaMnHCF@10%CNT different mass loadings, (**c**) NaMnHCF and NaMnHCF@10%CNT different mass loadings and (**d**) Logarithm of the chemical diffusion coefficient of Na^+ as a function of stoichiometry calculated from GITT.
